# Evolution of the Microstructure of PP-LDHs Nanocomposites during Melt Compounding: A Simulation Approach

**DOI:** 10.3390/polym16010070

**Published:** 2023-12-25

**Authors:** Giulia Bernagozzi, Rossella Arrigo, Alberto Frache

**Affiliations:** 1 Department of Applied Science and Technology, Politecnico di Torino, Viale Teresa Michel, 15121 Alessandria, Italy; giulia.bernagozzi@polito.it (G.B.); alberto.frache@polito.it (A.F.); 2 INSTM Local Unit, 50121 Firenze, Italy

**Keywords:** polypropylene, layered double hydroxides (LDHs), rheology, melt compounding, simulation

## Abstract

In the context of polymer-based nanocomposites containing layered nanofillers, the achievement of good extents of dispersion and distribution of the embedded nanoparticles and, even more, the obtainment of intercalated and/or exfoliated structures through melt compounding still represents a persistent challenge, especially in the case of anionic layered double hydroxides (LDHs)-containing systems and non-polar polymeric matrices. In this work, a simulation approach is proposed to evaluate the influence of the processing conditions on the morphology of polypropylene (PP)-based nanocomposites containing organomodified LDHs. In particular, the effect of the screw rotation speed and the feed rate on the final microstructure of the materials formulated through melt compounding in a twin-screw extruder was assessed. The rheological and morphological characterizations demonstrated that a more homogeneous morphology was achieved when high levels of both exploited processing parameters are selected. The results coming from the simulation of the processing were used to establish some relationships between the flow parameters and the microstructure of the nanocomposites, demonstrating that low residence times coupled with high local shear rates are required to ensure the achievement of homogenous morphologies, likely involving the occurrence of intercalation phenomena.

## 1. Introduction

In the last decades, the interest towards polymeric nanocomposites has continuously increased, mainly due to their remarkable improved structural and functional properties as compared to their unfilled counterparts [1,2,3]. Importantly, through the incorporation of different kinds of nanofillers, a widening of the typical field of application of polymeric materials can be achieved, owing to the enhancement of the mechanical, barrier, optical, electrical and thermal properties [4,5]. In particular, several studies on polypropylene (PP)-based nanocomposites reported in the literature demonstrated that the embedded nanofillers can provide the matrix with superior flame retardancy [6,7] and mechanical performances [8,9]. Among the different types of nanofillers exploited for the formulation of such kinds of nanostructured materials, nanoclays have a prominent role and extensive investigations have highlighted their importance [10].

Obviously, the final properties of polymer-based nanocomposites depend on the kind of embedded nanofiller, the shape and the aspect ratio, as well as on the quality of the polymer/filler interface which, in turn, is governed by the interactions established between the two components [11,12,13]. The key to attaining optimal mechanical properties lies in effectively controlling the morphology of the nanocomposites, particularly through the precise distribution and dispersion of the filler within the polymer matrix [1,10]. The ultimate morphology of a nanocomposite obtained through melt compounding is influenced by various thermodynamic and kinetic factors, including interfacial tension, viscosity, shear stress and processing conditions, among few to mention [14,15,16]. In this context, when nonpolar polymers (such as polypropylene) were selected as the matrix, the achievement of a good extent of dispersion of hydrophilic inorganic fillers has proven to be a persistent challenge [17,18]. Additionally, several studies have demonstrated that all the described issues related to the difficult dispersion of inorganic nanofillers within nonpolar matrices are further exacerbated for layered double hydroxides (LDHs), namely hydrotalcite, belonging to the class of cationic clays [19,20].

The most commonly used strategy to improve polymer/nanofiller compatibility involves the use of compatibilizers, such as copolymers containing a functional group able to interact with the surface of the inorganic particles [21,22,23,24]. Another solution for ameliorating the morphology of LDHs-containing systems involves the modification of the starting hydrotalcite with organic functionalities, such as the oleate group [7,8], which are positioned in the interlayer galleries, causing an increase in the distance between the platelets. In such a way during the melt processing, the polymer can enter between the layers, facilitating the formation of intercalated/exfoliated hybrids.

Besides the chemical modifications that can enhance the dispersion and distribution of a nanofiller within a polymeric matrix, the selection of the processing parameters during the melt compounding step plays an important role [25]. Typically, twin-screw extruders are the most used processing apparatuses to formulate polymer-based nanocomposites, mainly due to the fact that all the processing parameters can be changed independently [26,27]. As an example, the screw speed and the feed rate can be set quite independently, and even the screw profile can be designed on purpose. Several studies are focused on the optimization of the processing conditions in order to obtain high extent of intercalation and exfoliation. As an example, the effects of the concentration of PP grafted with maleic anhydride (PP-g-MA) as compatibilizer and nanoclays, and the screw speed on the extent of exfoliation, have been evaluated, documenting the obtainment of better performances for high content of PP-g-MA [28]. Additionally, the effects of the screw profile on the microstructure of PP/organoclay nanocomposites were evaluated, demonstrating that the level of intercalation is not affected by the processing conditions, while the exfoliation depends on the cumulative deformation, the screw profile and the feed rate [29,30,31]. Furthermore, an enhancement of the mechanical properties was observed when the shear stresses during melt-compounding were maximized [21]. Other authors studying PP/carbon nanotube nanocomposites confirmed that the screw profile can be designed on purpose as a function of the final properties [32].

Besides the experimental approach, the use of simulation software allows for significantly reducing the number of extrusions to be made, in order to optimize in less time and with lower costs the melt compounding of nanocomposites. To this aim, the software Ludovic^®^ (V7.1), developed by B. Vergnes et al., in 1998 [33], can perform flow simulations by varying the screw profile, the temperature profile, the screw speed and the feed rate, obtaining several flow parameters that can be related to the dispersion/distribution of a nanofiller. Several authors have effectively proven that Ludovic^®^ software is able to correctly simulate the flow conditions within a twin-screw extruder [34,35,36,37,38,39]. However, to the best of the authors’ knowledge, this approach has not been used for evaluating the effect of the processing conditions and for optimizing the process parameters on LDHs-containing nanocomposites.

In this work, PP-based nanocomposites containing 5 wt.% of organomodified LDHs were formulated through melt compounding using a twin-screw extruder. Different combinations of screw speed and feed rate were exploited, following the results of the DoE analysis performed with Ludovic^®^ Software. The effects of the different processing conditions on the final morphology of the nanocomposites were assessed through rheological and morphological characterizations, also evaluating the evolution of the material microstructure along the screw. Finally, the obtained results were related to the flow parameters derived by the Ludovic^®^ simulations, allowing for disclosing important relationships between the residence time, the shear rate and the microstructure of the nanocomposites.

## 2. Materials and Methods

### 2.1. Materials

A homopolymer PP (Moplen HP500N supplied by Lyondellbasell, Rotterdam, The Netherlands) with MFR of 12 g/10 min (230 °C/2.16 kg) was used as the matrix. A PP grafted with maleic anhydride (0.6 wt.%, supplied by Sigma-Aldrich (Darmstadt, Germany), hereinafter named PP-g-MA) was used as a compatibilizer. Layered double hydroxides (LDHs) modified with oleate groups (o-LDHs, [Mg_0.66_Al_0.34_(OH)_2_](C_18_H_33_O_2_)_0.34_ × H_2_O, D50 = 3.78 µm, D90 = 10.83 µm t) were supplied by Prolabin&Tefarm (Perugia, Italy).

### 2.2. Methods

#### 2.2.1. Flow Modeling

The melt flow along the extruder was simulated using Ludovic^®^ software, which allows the calculation of different melt parameters, such as temperature, pressure, viscosity, shear rate and residence time, using a local one-dimensional approach [33]. The simulated process is described under stationary conditions, and the results are along the axial direction of the screw. These simplifications may result in a fairly large margin of error compared to the real case. However, several experimental applications have shown the software’s ability to adequately represent the flow conditions within an extruder [39,40].

In this work, Ludovic^®^ was firstly used to perform a DoE (Design of Experiments) analysis, that can execute a very large number of simulations giving as input a range of values. For carrying out the DoE analysis, a preliminary thermal and rheological characterization of the polymer was necessary to meet the software requirements. In particular, the thermal characteristics of the material (i.e., melting temperature and enthalpy) were measured through DSC analyses (using a DSC Q20 apparatus, TA Instruments (New Castle, DE, USA)) by performing the following thermal cycle: a first heating scan from −50 °C to 200 °C, a cooling scan from 200 to −50 °C and a second heating ramp from −50 °C to 200 °C, keeping the heating/cooling rate at 10 °C/min. The rheological data (flow curves at three different temperatures) were obtained by performing frequency sweep tests (ARES rheometer, TA Instrument (New Castle, DE, USA), considering a strain amplitude within the linear viscoelastic region of the samples, shear rate ranging from 0.1 to 100 1/s, and temperatures of 190, 210 and 230 °C. All the rheological measurements were carried out under a nitrogen atmosphere to avoid material degradation. The heat transfer coefficient between the polymer and the barrel was fixed at 350 W/m^2^K, according to what was reported in the literature for PP [41].

**Figure 1:**
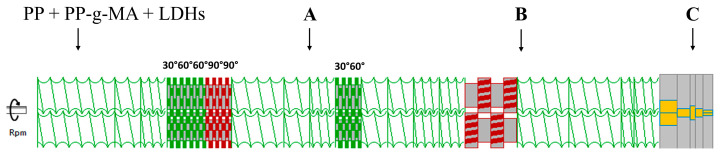
Screw profile and indications of the different zones in which the specimens for the further characterizations were collected.

To simulate the melt compounding process, a co-rotating twin-screw extruder Leistritz ZSE 18 HP (Nuremberg, Germany) having a screw diameter of 18 mm, barrel length of 720 mm and L/D = 40 was used. The selected screw configuration, reported in Figure 1 and Table 1, involves conveying elements and three mixing zones with different elements to promote dispersive/distributive mixing. In particular, a first kneading block is present in order to plasticize the polymer and incorporate the filler; then after a conveying zone, a second kneading block with lower stagger angles is present. Finally, at two-thirds of the screws, a toothed mixing element (TME) was inserted to homogenize the material before the further conveying zone up to the die.

The DoE analysis was performed (considering the constraints of both the available extruder and the material to be processed) by selecting the following inputs (summarized in Table 2): feed rate from 1 to 6 kg/h with intermediate steps of 0.5 kg/h, screw rotation speed from 100 to 400 rpm with intermediate steps of 50 rpm, temperature from 190 to 220 °C with intermediate steps of 10 °C.

Since the results obtained from the analysis of the response surfaces demonstrated that the temperature profile does not significantly affect the flow conditions (likely due to the low thermal sensibility of the material viscosity in the range of shear rate involved during the processing), the variation of this parameter was not considered for the subsequent simulations.

The results of the DoE analyses were then analyzed taking into consideration the influence of the screw speed and the feed rate on the following processing characteristics: shear rate, torque, total residence time, cumulated strain, specific mechanical energy, pressure, actual temperature and actual viscosity along the screws. For each of the evaluated outputs, the surface responses as a function of the two exploited processing variables (i.e., screw speed and feed rate) were analyzed (see representative graphs reported in Figure S1), aiming at selecting the processing conditions for the experimental formulation of the nanocomposites.

Ludovic^®^ software was further exploited for the computation of the residence time, the shear rate and the cumulated strain along the screw at the actual processing conditions selected for the experimental processing of the nanocomposites (as detailed in Section 2.2.2), aiming at correlating the data obtained through the experimental characterizations and the simulation results.

#### 2.2.2. Processing of PP-LDH Nanocomposite

The nanocomposites were prepared using the already described co-rotating twin-screw extruder Leistritz ZSE 18 HP (Nuremberg, Germany), equipped with two gravimetric feeders (one dedicated to the polymer pellets and the other for the filler powder). The screw configuration is reported in Figure 1 and Table 1 and detailed in the previous paragraph.

For the formulation of the nanocomposites, 5 wt.% of LDH and 5 wt.% of PP-g-MA were introduced within PP. All the materials were fed together in the hopper. The unfilled matrix containing 5 wt.% of PP-g-MA was subjected to the same processing.

Different melt compounding processes were performed, by varying the processing condition on the basis of the results of the DoE analysis: temperature at 190 °C for all the materials, screw speed at 150 or 350 rpm and feed rate at 2 or 4 kg/h. After each processing, the extrudate was collected, cooled in a water bath and pelletized. Besides, aiming at investigating the evolution of the microstructure of the nanocomposite along the screws, specimens were also collected in two different zones along the extruder barrel (zones A and B shown in Figure 1). The as-collected samples were immediately cooled in water after their sampling.

Table 3 reports the sample codes used hereinafter. Each specimen has been named adding the screw speed and the feed rate of the extrusions and the zone in which it was collected. In the table, PP_ref refers to the unfilled PP/PP-g-MA system (5 wt.% of compatibilizer) processed at 150 rpm and 2 kg/h.

#### 2.2.3. Characterization Techniques

Specimens intended for rheological characterization have been produced by compression molding by using a hot-plates press Collin P (Maitenbeth, Germany). The specimens (25 mm diameter, 1 mm thickness) were obtained at 190 °C, with a pressure of 100 bar for 2 min.

Rheological analyses have been carried out by means of an ARES (TA Instrument, New Castle, DE, USA) strain-controlled rheometer in parallel plate geometry. Complex viscosity values were measured by performing dynamic frequency sweep tests at 190 °C from 0.01 to 100 rad/s under a nitrogen atmosphere.

EVO 15 Scanning Electron Microscope (SEM, beam voltage: 20 kV; working distance: 8.5 mm) by Zeiss (Oberkochen, Germany) was used to carry out the morphological characterization of the samples and to verify the dispersion and distribution of the nanofiller. The specimens (either extrudates or samples collected in zones A and B) were fractured in liquid nitrogen, then the obtained surfaces were covered with a sputtered gold layer and analyzed.

## 3. Results

### 3.1. Effect of the Processing Conditions on the Nanocomposite Morphology

The rheological behavior of nanocomposites is widely used to characterize the state of dispersion and/or distribution of the embedded nanofiller within the host matrix, as well as to disclose the possible polymer/polymer, polymer/filler, and filler/filler interactions established in such kinds of nanostructured materials. Therefore, small-amplitude oscillatory shear measurements were carried out on the nanocomposites processed using the different combinations of processing parameters, aiming at assessing the possible effect of the processing on the material microstructure.

**Figure 2:**
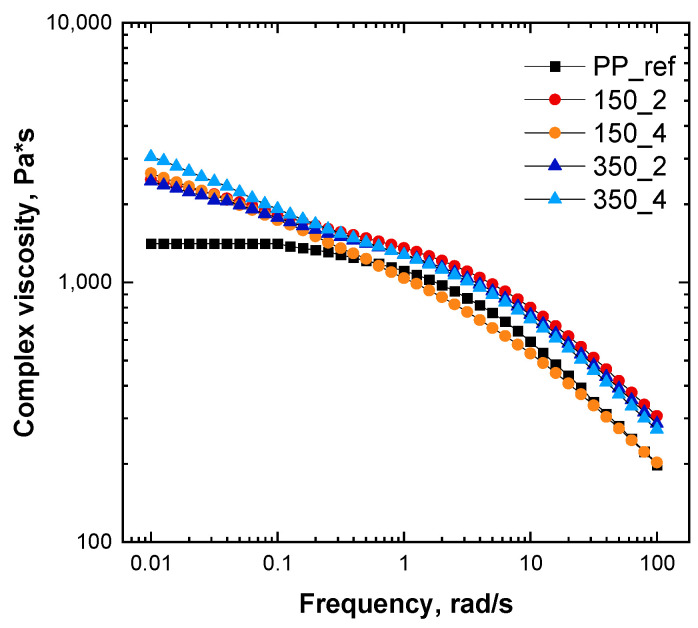
Complex viscosity curves of PP and PP/LDH nanocomposites processed at different conditions.

Figure 2 reports the complex viscosity as a function of the frequency for all the formulated nanocomposites (the curves refer to the extrudates, collected after the extruder die). As expected, unfilled PP shows a typical Newtonian behavior at low and intermediate frequencies followed by a mild shear thinning in the high-frequency region. The introduction of the LDHs, irrespective of the used processing conditions, causes the disappearance of the matrix Newtonian plateau and a general amplification of the non-Newtonian features. In particular, in the low-frequency region, the appearance of a yield stress behavior, involving a steep rise of the complex viscosity when the frequency decreases, is clearly observable for all the systems. As widely reported in the literature, the appearance of the yield stress behavior can be ascribed to a limited motion of the polymer macromolecules that, interacting with the embedded nanofillers, are no longer able to fully relax [9,19,42]. Additionally, from the complex viscosity curves depicted in Figure 2, it is clearly observable that the effect of the embedded nanofillers on the PP rheological behavior is more pronounced in the low-frequency region, where the rheological response of a large portion of macromolecules is recorded. At variance, the rheological response at high frequencies, reflecting the relaxation dynamics of portions of the chain between the entanglements, is almost governed by that of the unfilled polymer.

Let us discuss the possible effect of the processing conditions on the rheological behavior and, hence, the morphology of the nanocomposites. As observable for the complex viscosity curves reported in Figure 2, the different processing parameters adopted during the melt compounding induce some differences in both low- and high-frequency responses. In particular, the dissimilar behavior recorded in the high-frequency region can be attributed to some orientation phenomenon, involving not only the polymer chains but also the embedded nanofillers, leading in the case of the system processed at 150 rpm at 4 kg/h the achievement of lower complex viscosity values as compared to the unfilled PP. Nevertheless, since the behavior recorded at such high frequencies is mainly governed by that of the matrix, more useful information about the state of dispersion of the embedded nanofillers can be gained by analyzing the low-frequency response.

**Figure 3:**
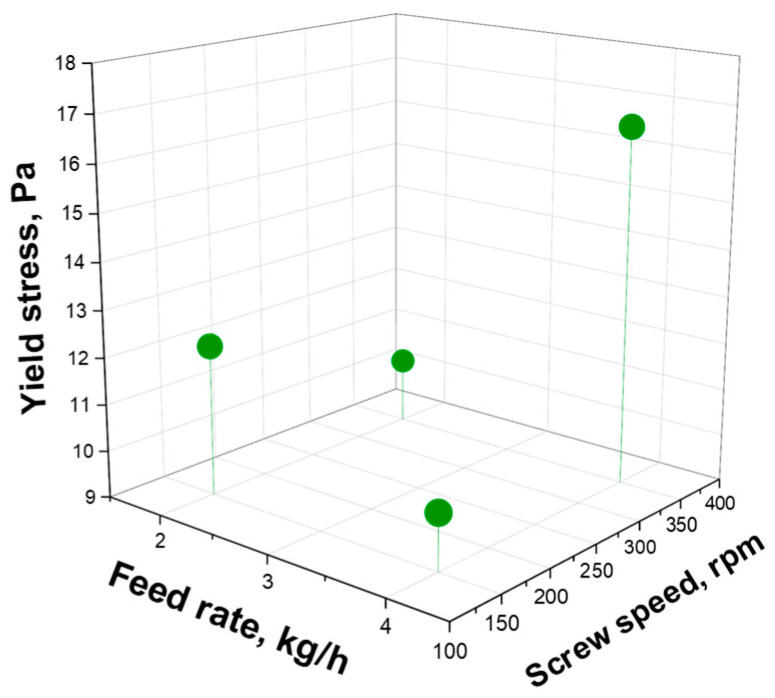
Effects of feed rate and screw speed on the yield stress.

As already discussed, all the formulated nanocomposites exhibit yield stress behavior, which, at fixed nanofiller content, can be related to the level of dispersion of the nanofillers within the polymer matrix, and thus also to the occurrence of possible melt intercalation/exfoliation phenomena. In other words, since the physical phenomena underlying the yield stress are related to the extent of polymer/filler interactions, this rheological property can be considered as a sort of qualitative measurement of the nanofiller dispersion [31,43]. To quantitatively evaluate the yield stress, the experimental complex viscosity data were fitted using the following model [44,45]:

(1) $$\eta^{*}\left(\omega \right)=\frac{\eta_{0}}{{\left[1+(\lambda \omega )\right]}^{\left(1-n\right)}}+\frac{\sigma_{0}}{\omega }$$

where σ_0_ is the melt yield stress, η_0_ is the zero-shear viscosity, λ is the relaxation time and n is the power law index. In Figure 3, the obtained values of yield stress for the different nanocomposites processed at the various screw speeds and feed rates are reported (the values of all the fitting parameters are listed in Table S1). From a general point of view, the highest value of yield stress was obtained for the nanocomposite processed at 350 rpm and 4 kg/h. However, a straightforward effect neither of the screw speed nor the feed rate cannot be recognized, since, when the processing is performed at 150 rpm, the yield stress decreases when the feed rate is increased. On the contrary, if the screw speed is set at 350 rpm, a higher yield stress value is obtained at a high feed rate.

**Figure 4:**
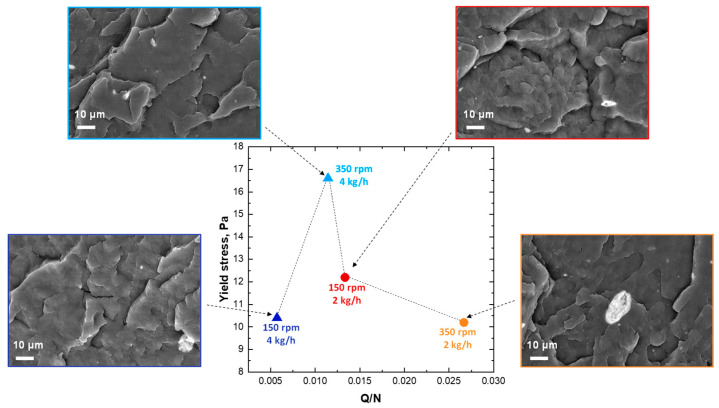
Evolution of yield stress and morphological observations as a function of Q/N (in the SEM micrographs; the polymeric matrix is in dark gray while white spots refer to the embedded LDHs).

Therefore, aiming at taking into consideration the combined effect of the two processing parameters, the values of yield stress were plotted as a function of the ratio Q/N (where Q is the feed rate and N is the screw rotation speed), which is usually exploited for the evaluation of the effect of the processing parameters during an extrusion process, being proportional to the fill ratio of the screw [36]. Figure 4 displays the evolution of the yield stress as a function of the ratio Q/N and, also in this case, a non-monotonic trend is obtained. More specifically, the highest value of yield stress is obtained at Q/N equal at 0.011, thus for higher feed rate and higher screw speed; a high extent of LDHs dispersion and distribution is thus expected for the system processed using these conditions. For the nanocomposites obtained using the other exploited combination of the processing parameters, lower values of yield stress are obtained, suggesting the worst dispersion of the nanofillers. The obtained results are in disagreement as compared to what was reported in the literature for exfoliated PP/nanoclays nanocomposites, for which a progressively decreasing trend of the yield stress as a function of Q/N was found [36]. However, it should be considered that, in the case of polymeric systems containing anionic layered double hydroxides, as in the study reported here, the achievement of considerable extent of dispersion of the nanofiller is more difficult than for traditional cationic nanoclays, given the very high values of the surface charge density of LDHs, which makes practically impossible from a thermodynamic point of view the obtainment of exfoliated structures through melt compounding [46].

The morphological analyses confirm what is inferred on the basis of the rheological measurement. In fact, the nanocomposite 350_4 (Q/N = 0.011) shows a quite uniform and homogeneous morphology, indicating that the nanofiller was successfully nanodispersed, although some clusters having submicrometric dimensions (about 2–5 μm) can be observed. The dispersion of the embedded LDHs for this nanocomposite was further characterized through EDX analysis. In particular, looking at the maps reported in Figure S2, it can be seen that both Al and Zn, hence LDHs, are uniformly present in the whole characterized sample. Differently, the SEM micrographs of the nanocomposites processed considering the highest Q/N demonstrated the presence of several bigger aggregates (having mean dimensions of about 10–50 μm), although EDX characterization (Figure S3) testified also for these systems the achievement of a satisfying nanofiller dispersion.

### 3.2. Evolution of the Nanocomposite Morphology along the Screws

**Figure 5:**
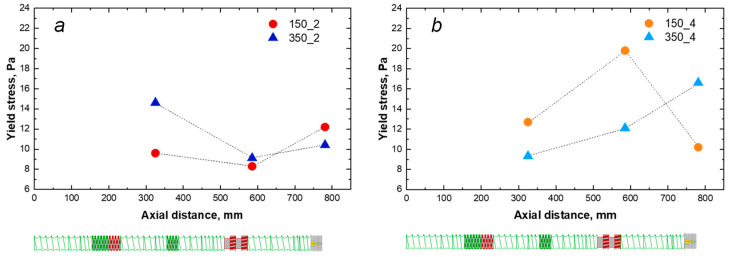
Effects of feed rate, screw speed and axial distance on the yield stress: (**a**) fixed feed rate 2 kg/h; (**b**) fixed feed rate 4 kg/h.

Aiming at assessing the evolution of the morphology of the nanocomposites along the extruder, in addition to the extrudates recovered at the die exit, two other samplings have been picked up in two different zones, as already explained in the previous section and illustrated in Figure 1. The specimens collected in zones A and B were tested through small-amplitude oscillatory shear measurements, and the experimental results, in terms of variation of the complex viscosity as a function of the frequency, were fitted with the model reported in Equation (1). Following the same approach as in the previous section, also in this case the calculated yield stress values were exploited as a qualitative index for assessing the dispersion and distribution of the LDHs within the host matrix. Figure 5 depicts the yield stress values as a function of the position along the screws.

Looking at the results reported in Figure 5a, referring to the nanocomposites processed at 2 kg/h, it can be observed that, from a general point of view, for both selected screw rotation speeds the yield stress tends to decrease after the second kneading block and the TME block and to recover after the die exit. In particular, after the first kneading block, the yield stress is higher when the nanocomposite is processed at 350 rpm, indicating that a better degree of nanofiller dispersion was achieved in these conditions in the first part of the screws. At variance, the decrease observed passing from zone A to zone B is less severe when the processing is carried out at 150 rpm. Finally, from zone B to the die exit, both screw speed values induce an increase in the yield stress values that, as already commented in the previous paragraph, is higher for the extrudate processed at 150 rpm.

The effect on the yield stress when the feed rate is set at 4 kg/h is reported in Figure 5b. Similarly to what was observed for the nanocomposites processed at 2 kg/h, a non-monotonic trend is obtained when the processing is carried out at 150 rpm, although in this case the yield stress shows a remarkable increase from A to B, and then a drop until the die exit. Differently, for the nanocomposite processed at a high feed rate and high screw speed, the yield stress progressively increases along the screws, leading to the achievement of the highest value at the die exit. Therefore, as already commented in the previous paragraph, generally the combination of processing parameters 350_4 results in a better dispersion/distribution of the nanofiller, and the evolution of the yield stress shows that the dispersion/distribution is gradually achieved along the screws. On the other hand, the combination of processing parameters 150_4 seems to promote a better dispersion/distribution along the barrel until zone B, although during the last section of the extruder, composed of conveying elements, a progressive worsening of the nanocomposite morphology is observed.

**Figure 6:**
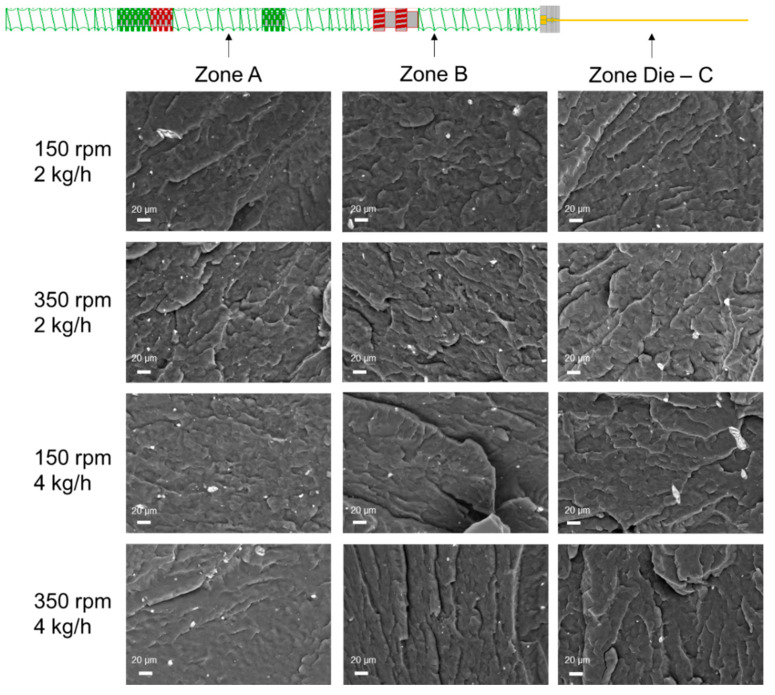
Morphological observations as a function of the processing parameters and the zone along the barrel (in the micrographs, the polymeric matrix is in dark gray while white spots refer to the embedded LDHs).

The morphological observations carried out with SEM can further clarify the trend of the yield stress and thus the dispersion/distribution of the nanofiller along the barrel. In Figure 6, the SEM micrographs of the nanocomposites processed using the different conditions and collected in the three zones along the barrel are reported. The nanocomposite processed at 150 rpm and 2 kg/h displays some aggregates in the zones along the barrel and a better dispersion when collected at the exit die, confirming the trend of the yield stress reported in Figure 5a. At fixed feed rate, the increase in the screw rotation speed promoted the achievement of a more uniform morphology in zone A, while some bigger clusters were observed proceeding along the screws. Interestingly, for the system 150_4, a higher extent of LDH distribution can be observed in zone B, as already inferred through the analysis of the rheological data. Finally, the selection of 350 rpm and 4 kg/h resulted in a gradually enhanced better dispersion/distribution as proceeding along the barrel, confirming also in this case the conclusions drawn by the trend of the yield stress.

### 3.3. Flow Parameters—Morphology Relationships

In the previous paragraphs, the effects of screw speed and feed rate on the morphology of the formulated nanocomposite were evaluated and discussed, taking into consideration the material rheological behavior and morphology, as well. Either for the extrudates collected after the melt compounding step or for the samples collected along the screw during the processing, non-monotonic trends as a function of the selected processing parameters were found, indicating the occurrence of different and concurrent phenomena during the processing (such as improved dispersion and melt intercalation or flocculation and reaggregation) which contribute to determining the final morphology of the nanocomposite. To gain a better understanding of the obtained results, the melt compounding process was simulated through Ludovic^®^ software, and the local shear rate and the residence time were selected as parameters to follow the state of dispersion of LDHs within the PP matrix.

**Figure 7:**
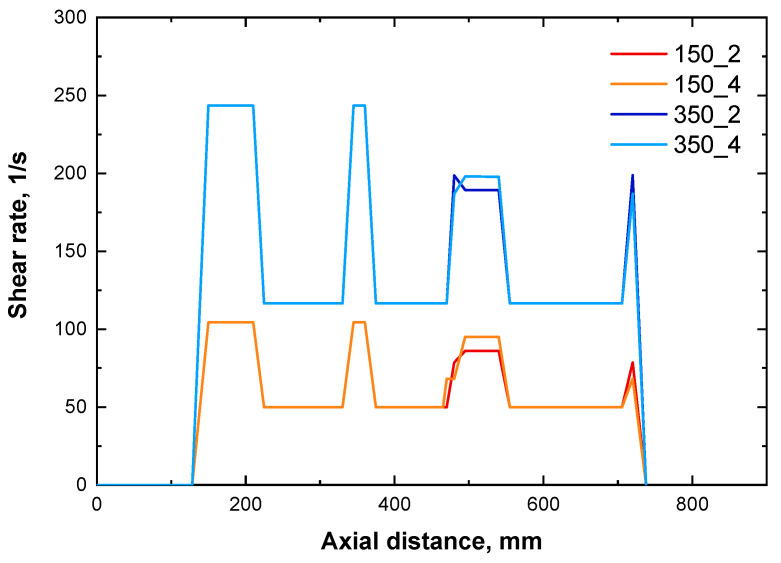
Evolution of the local shear rate along the barrel for the different processing conditions.

Figure 7 displays the evolution of the shear rate along the screws for the different selected combinations of processing parameters. As expected, the local shear rate increases in any position when the screw rotation speed is increased from 150 to 300 rpm. Furthermore, irrespective of the processing conditions, an increase in the shear rate values can be noticed in correspondence of the kneading and TME blocks. At fixed screw speed, the evolution of the shear rate is not affected by the feed rate, except for small variations in proximity of the TME elements.

Table 4 reports the values of the minimum residence time, taking into account that, according to the literature, this value is very similar to that experienced by most of the polymer chains [47]. At fixed screw speed, an increase in the feed rate causes a decrease of the residence time; at variance, at constant feed rate, the residence time decreases when the screw speed increases. However, it can be observed that the variation of the feed rate induces more significant variation as compared to the screw speed; furthermore, higher the feed rate, lower the influence of the screw rotation speed on the residence time. Aiming at achieving a good dispersion and distribution of a nanofiller, the residence time must be high enough to allow the nanofiller to be dispersed within the polymeric matrix, although reaggregation phenomena are usually observed if the molten material spends a too long time inside the extruder.

**Figure 8:**
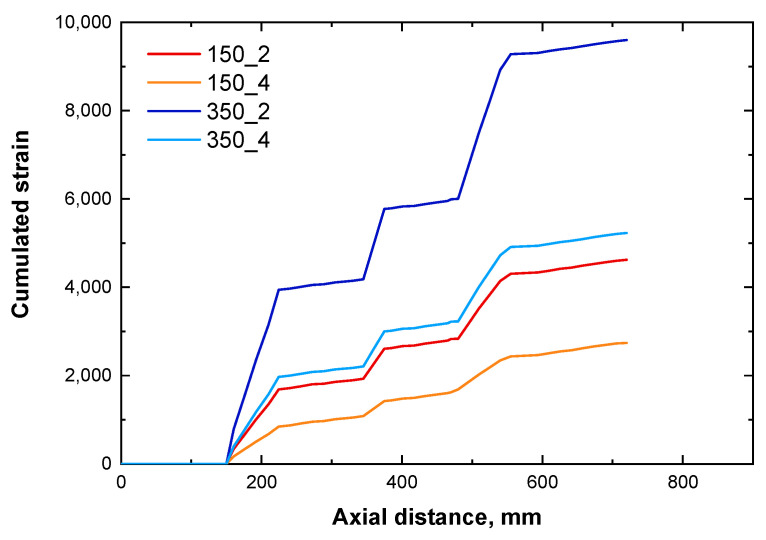
Evolution of the cumulated strain as a function of the processing parameters along the barrel.

In order to associate the morphology of the nanocomposites to the flow parameters, the cumulated strain (which is a linear combination of the local shear rate and the residence time) was further considered. As observable in Figure 8, the trend of the cumulated strain is similar for all the considered combinations of the processing parameters; more specifically, it increases in correspondence of the mixing blocks and remains almost constant along the zones of the screws containing conveying elements. Furthermore, higher values of the cumulated strain are obtained with increasing the screw speed and at low feed rates.

The results of the rheological measurements and of the morphological characterization, demonstrated the achievement of a more uniform morphology for the nanocomposite processed at 350 rpm and 4 kg/h. Furthermore, the analysis of the yield stress and of the morphology along the screw, demonstrated for this sample a progressive amelioration of the microstructure. On the other hand, the results obtained through the Ludovic^®^ simulations indicated that processing at high feed rate and screw speed involved the lowest residence time of the material inside the extruder and the highest values of local shear rate (resulting in cumulated strain values that are intermediate between the four combinations of parameters). Therefore, it can be inferred that high shear rates are required to achieve a good level of dispersion and distribution of the embedded LDHs (or also the formation of intercalated structures), along with short residence time to avoid reaggregation phenomena of the nanofillers within the extruder. In fact, the nanocomposite 350_2, which was subjected to the same shear rate than 350_4 but for a longer time, shown the presence of micrometric agglomerates of nanofillers, and a less uniform morphology as compared to the system processed at 4 kg/h. Therefore, although the cumulative deformation generated at 350 rpm and 2 kg/h is more important than in other conditions, the long residence time negatively affects the achievement of a uniform morphology, likely due to some reaggregation of the embedded nanofillers during the processing. In particular, looking at the evolution of the yield stress along the screws for this system (Figure 5a), it can be inferred that the supposed nanofiller reaggregation mostly occurs in the second part of the screws, after the second kneading block.

Finally, for the nanocomposites processed at low screw speed (i.e., 150 rpm), because of the low values of the local shear rate experienced by the material during the processing, the obtained results suggest that longer residence times are required to achieve a good extent of nanofiller dispersion. In fact, for the sample 150_2, involving the highest value of the residence time, a higher values of yield stress (hence, higher extent of LDHs dispersion) was recorded, as compared to 150_4. For this last sample, low local shear rate and low residence time, resulting in the lowest cumulated strain, did not allowed the achievement of a uniform microstructure, since SEM analyses demonstrated the presence of nanofiller clusters.

In all, from the analysis of the cumulated strain it appears that the optimal processing conditions have to be selected in order to reach a trade-off between the local shear rate and the residence time. In fact, the obtained analyses demonstrated that the nanocomposites 150_2 and 350_4, for which high values of yield stress (hence, better extent of LDHs dispersion/distribution) were achieved, underwent intermediate deformation during processing.

## 4. Conclusions

In this work, the effect of the processing parameters on the final morphology of PP-LDHs nanocomposites obtained through melt compounding in a twin-screw extruder is evaluated. In particular, the processing conditions were selected after a DoE analysis performed through the use of Ludovic^®^ software, which is able to simulate melt compounding processing. After the characterization of the rheological behavior and the morphology of the nanocomposites obtained using two different levels of screw rotation speed and feed rates, the experimental results were related to data coming from the simulation procedure, also considering the evolution of the material microstructure along the screws. The approach that was followed allowed for disclosing some relationships between the processing conditions, the flow parameters and the final morphology of the formulated nanocomposites, demonstrating that high shear stresses and low residence times (resulting in intermediate values of cumulative deformation) are required to achieve a good extent of LDH dispersion and distribution and the formation of intercalated or exfoliated structures.

## Figures and Tables

**Table 1:** Screw profile elements from the hopper to the die.

Length (mm)	Screw Elements
120	Conveying (pitch 30 mm)
30	Conveying (pitch 20 mm)
15	Kneading (angle 30°)
30	Kneading (angle 60°)
30	Kneading (angle 90°)
90	Conveying (pitch 30 mm)
30	Conveying (pitch 20 mm)
15	Kneading (angle 30°)
15	Kneading (angle 60°)
60	Conveying (pitch 30 mm)
60	Conveying (pitch 20 mm)
60	TME (angle 30°)
120	Conveying (pitch 30 mm)
45	Conveying (pitch 20 mm)

**Table 2:** Processing parameters and relative ranges exploited as inputs for the DoE analysis.

Input Parameter	Range	Intermediate Steps
Feed rate	1–6 kg/h	0.5 kg/h
Screw rotation speed	100–400 rpm	50 rpm
Temperature	190–220 °C	10 °C

**Table 3:** Sample codes as a function of the processing conditions.

Sample Code	Screw Speed (rpm)	Feed Rate (kg/h)	Collecting Zone
PP_ref	150	2	Die—C
150_2_A	150	2	A
150_4_A	150	4	A
350_2_A	350	2	A
350_4_A	350	4	A
150_2_B	150	2	B
150_4_B	150	4	B
350_2_B	350	2	B
350_4_B	350	4	B
150_2	150	2	Die—C
150_4	150	4	Die—C
350_2	350	2	Die—C
350_4	350	4	Die—C

**Table 4:** Residence times using the different processing conditions.

Sample Code	Residence Time (s)
150_2	109
350_2	86
150_4	59
350_4	50

## Data Availability

Data are available upon request.

## Supplementary Materials

The following supporting information can be downloaded at: https://www.mdpi.com/article/10.3390/polym16010070/s1, Table S1: Fitting parameters (Equation (1)); Figure S1: Main results of the DoE analysis performed through Ludovic^®^ software. Response surface for (A) shear rate, (B) total residence time, (C) torque and (D) cumulated strain as a function of screw speed and feed rate; Figure S2: EDX map for Al and Zn of 350_4; Figure S3: EDX map for Al and Zn of 150_4.
