# Supplementary material for: Evolution of the Microstructure of PP-LDHs Nanocomposites during Melt Compounding: A Simulation Approach
Source: Polymers (Basel). 2023 Dec 25;16(1):70. doi: 10.3390/polym16010070 (PMC10780429; doi:10.3390/polym16010070)
Supplement: Supplementary file 1 [file polymers-16-00070-s001.zip › polymers-2776512-supplementary.pdf]

Supporting Information

Evolution of the Microstructure of PP-LDHs Nanocomposites during Melt Compounding: A Simulation Approach

Giulia Bernagozzi [1], Rossella Arrigo[1,2,*] and Alberto Frache[1,2]

[1] Department of Applied Science and Technology, Politecnico di Torino, viale Teresa Michel Alessandria, Italy; giulia.bernagozzi@polito.it (G.B.), rossella.arrigo@polito.it (R.A.), alberto.frache@polito.it (A.F.)
[2] INSTM local unit
* Correspondence: rossella.arrigo@polito.it

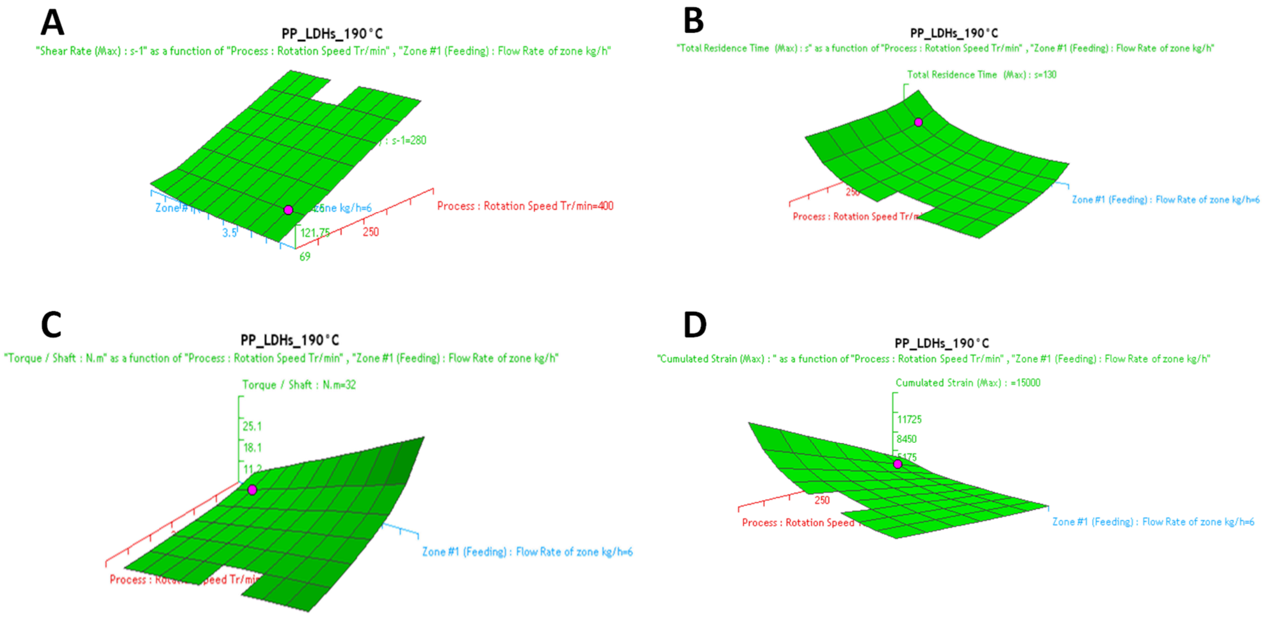

Figure S1. Main results of the DoE analysis performed through Ludovic® software. Response surface for (A) shear rate, (B) total residence time, (C) torque and (D) cumulated strain as a function of screw speed and feed rate.

Table S1. Fitting parameters (Equation 1).

Sample code	η_0 (Pa*s)	λ (1/s)	n	σ_0 (Pa)
150_2_A	1239.1	0.55	0.62	9.6
150_2_B	1127.5	0.41	0.60	8.3
150_2	1681.6	0.56	0.59	12.2
150_4_A	1586.5	0.44	0.60	12.7
150_4_B	1251.5	0.53	0.61	19.8
150_4	1784.3	3.03	0.63	10.4
350_2_A	1044.2	0.65	0.62	14.6
350_2_B	1168.2	0.60	0.65	9.1
350_2	1651.2	0.70	0.60	10.2
350_4_A	1393.2	0.42	0.61	9.3
350_4_B	1376.3	0.53	0.61	12.1
350_4	1747.2	1.02	0.61	16.6

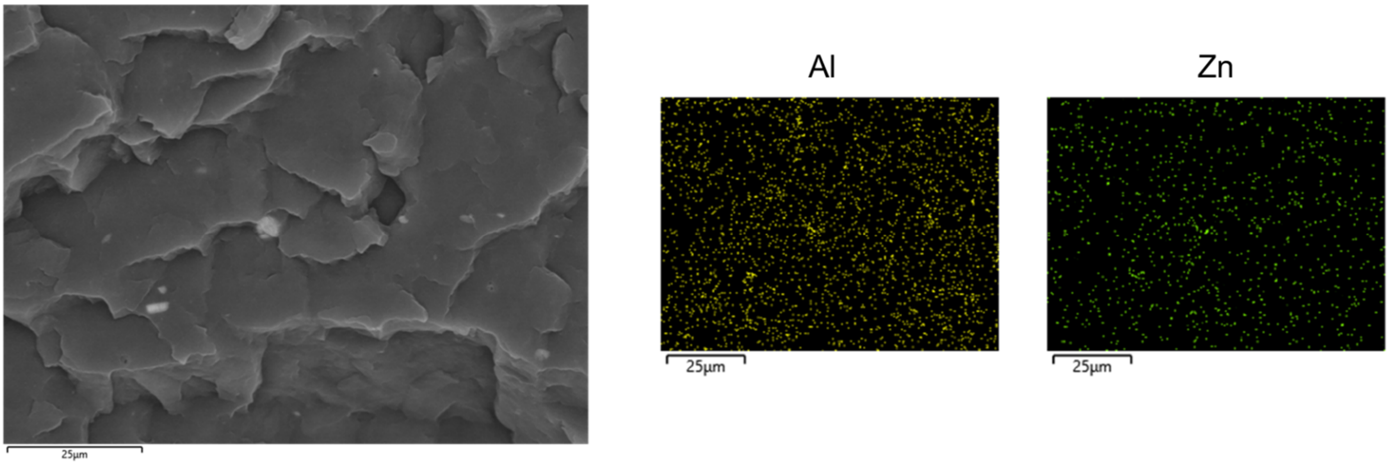

Figure S2. EDX map for Al and Zn of 350_4.

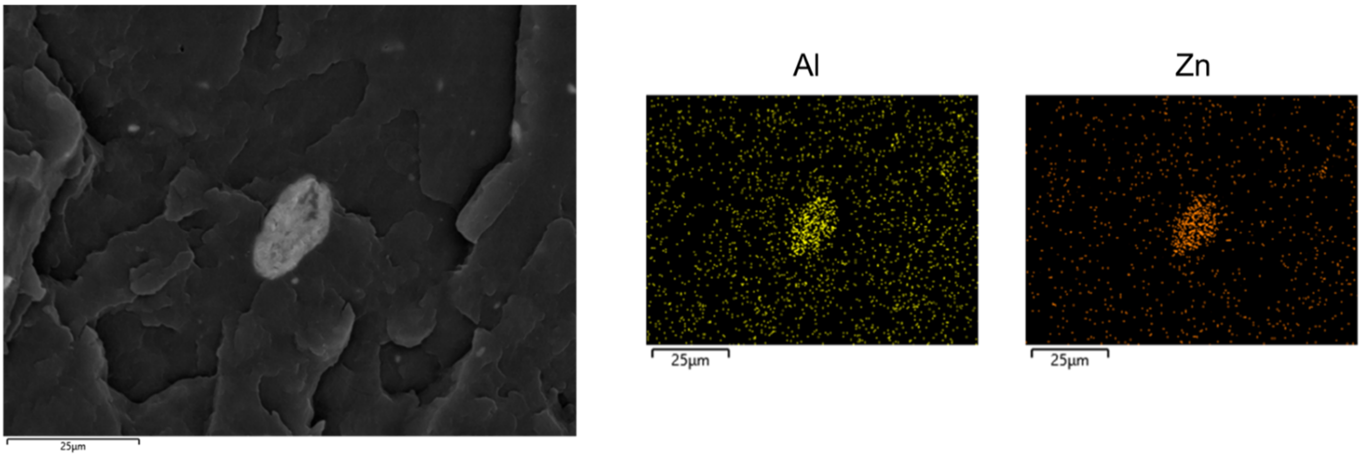

Figure S3. EDX map for Al and Zn of 350_2.
